# Transobturator tapes are preferable over transvaginal tapes for the management of female stress urinary incontinence: For

**DOI:** 10.4103/0970-1591.57905

**Published:** 2009

**Authors:** N. Rajamaheshwari, Lilly Varghese

**Affiliations:** Department of Urogynecology, Institute of Social Obstetrics, Government Kasturba Gandhi Hospital, Madras Medical College, Triplicane, Chennai - 600005, India; 1 Department of Obstetrics and Gynecology, Unit 1, Christian Medical College and Hospital, Ida, Vellore, India

**Keywords:** Stress urinary incontinence, tension free, treatment, transobturator tape, transvaginal tape

## Abstract

Tension-free midurethral slings have proven to have low morbidity and high success rates in the management of female stress urinary incontinence. Among midurethal slings, the transobtuator tapes has comparable success and satisfaction rates as the transvaginal tapes but with reduced risk of intra-operative bladder injury, shorter operating time and quicker return to activities. Thus, the transobturator tapes may be recommended as the primary choice for the treatment of female stress urinary incontinence.

The minimally invasive synthetic midurethral sling (transvaginal tape or TVT) has become the preferred surgical method for the treatment of female stress urinary incontinence (SUI) since its introduction in 1995 by Ulmsten U *et al*.[1] Despite its minimally invasive nature and enduring success rates, the blind passage of trocars along the retropubic route for placement of this sling isknown to cause inadvertent vesical injury and life-threatening complications such as injury to the iliac vessels and bowel.

The alternate transobturator route (outside-in) for application of the midurethral sling was developed by Delerome in 2001[2] to reduce these complications. This sling and its modification as the inside-out technique by Jean de Leval in 2003[3] have gained widespread acceptance. Serious complications are lower with the transobturator (TOT) route and this has minimized the need for peroperative cystoscopy. Over time the TOT has proven to be a safe procedure and in order to establish its superiority over TVT, we review the literature assessing these two procedures in terms of efficacy, operative complications, voiding dysfunction, return to normal activity and long-term complications.

## EFFICACY

The TVT has proven to be a very effective procedure both in the short- and long-term follow-up studies. It has similar 5-year cure rates as the Burch colposuspension, which was originally considered to be the gold standard for female SUI.[4] A Nordic study with the longest follow-up to date showed that there is no decline in efficacy of the TVT procedure over time (11 years) with 90.2% objective cure rate.[5] The 5-year post-treatment success rates for TVT have been confirmed to be 84.5% for pure SUI, 67% for the mixed incontinence cases, and 72.2% for cases when associated with sphincter deficiency.[6] It is therefore necessary to compare the success rates of the relatively newer TOT with the TVT.

Comparative multicentre randomized control trials (RCTs) evaluating the efficacy of TOT showed similar objective cure rates as the TVT at 3 months,[7] at 6 months[8] and 1 year (complete cure rate of SUI was 91%).[9] In a systematic review and meta-analysis of effectiveness and complications of TOT versus TVT, Latte *et al*. assessed 11 RCTs including 1261 women. They showed equivalent cure rates in the TOT group vs TVT (OR: 1.05; 95% CI: 0.64-1.70).[10]

Ulmsten *et al*. quoted 3-year cure rates for the TVT; 86% of the women were completely cured and another 11% were significantly improved.[11] The midterm success rates for the TOT are also similar, with a minimum 3-year follow-up showing a cure rate of 88.4% and an improvement in a further 9.3% of the patients.[12]

The efficacy and cure rates of the TOT therefore appear to be equivalent to that of the TVT at short- and midterm follow-up.

Araco *et al*. classified women with SUI based on its symptomatic severity and the urodynamic evaluation as SUI 1 and SUI 2 (SUI 1 = loss of urine during excessive strains, SUI 2 = during minor strain). They assessed the comparative efficacy of TVT vs TOT in these groups of women and found that SUI 1 patients treated with TOT were 100% cured, while only 66% of patients with SUI 2 were cured. TVT on the other hand provided 100% cure in both groups of SUI. Though Araco *et al*. recommended TOT for milder grades of SUI and TVT for the more severe grades of SUI, further well-powered studies and longer follow-ups are required to clarify this issue.[13]

## INTRAOPERATIVE COMPLICATIONS

A recent review revealed the total reported complication rates ranging from 4.3% to 75.1% for TVT and from 10.5% to 31.3% for TOT.[14] There is a statistically significant increased intraoperative risk of bladder perforations and hematomas with TVT where the retropubic route is used. Serious complications such as bowel injury, major vascular injury and death can occur with the TVT.[14] In contrast, no bladder, urethral injuries and vascular trauma (hematoma or bleeding) occurred in a study by de Leval *et al*.[3] who introduced the inside-out TOT.

Various multicenter RCT's[7,8] and systematic reviews[10,15] are in accord, with intraoperative bladder perforation rates varying from 0%[7,8] for TOT and 4%[8] to 8.5[7] for TVT. In a systematic review, Latte *et al*. reported the odds for bladder injury (OR 0.13; 95% CI 0.06-0.27) were less for all TOT tapes.[10] Similarly, the results of Novara *et al*. in their systematic review comparing retropubic and TOT tapes showed that occurrence of bladder perforations, pelvic hematoma and storage lower urinary tract symptoms (LUTS) were significantly less common in the patients treated by TOT.[15]

de Leval *et al*. opined that, unlike the TVT, intraoperative cystoscopy is not required while performing the TOT procedure as the TOT tape does not enter the pelvis.[3] Intraoperative cystoscopy confirmed the absence of bladder perforation in the first 40 cases in their series of 102 patients who underwent the TOT procedure.[9]

Waltregny reviewed 30 studies, which reported complications during the TOT procedure. Only one intraoperative urethral (0.02%) and two (0.04%) bladder injuries were diagnosed among 5,385 patients.[16]

The Austrian TOT registry evaluated 2,543 procedures (using both TOT approaches) and confirmed that bladder (0.4%) and urethral (0.1%) perforations are rare with this approach.[17] Similarly, a retrospective analysis by Abdel-Fattah *et al*. on the incidence of lower urinary tract injuries in 389 women who underwent TOT found that injuries occurred in women who underwent concomitant reconstructive vaginal surgeries and secondary procedures. They concluded that lower tract injury is an uncommon complication of the TOT procedures and that intraoperative cystoscopy may be considered in selected cases.[18]

The TOT thus scores heavily over the TVT in avoiding these major issues.

## VOIDING DYSFUNCTION

De novo occurrence of voiding dysfunction is another major area of concern following the insertion of midurethral slings.

Two multicenter RCTs done in Australia and Italy, which investigated perioperative complication are in agreement that early postoperative urinary retention rates, voiding difficulty and urinary tract infection were similar for both the retropubic and the TOT approaches.[7,8] Takedown (tape incision or removal) rates for TVT and TOT are also similar. In a series comparing the effect of TVT vs TOT on SUI and mixed urinary incontinence, 3.6% of the TVT group required takedown while there were none in the TOT group.[19] Take down rate following TOT was reported to be 2.8%[3] to 3.9%.[9]

Women who undergo TVT and TOT have a significant improvement in LUTS and voiding function postoperatively. However, the rate of de novo urgency after TVT is 5.9% to 25%,[14] which is higher than that seen after the TOT procedure occurring in 0% to 13.9% at 1 year follow-up.[16] The voiding dysfunction is expectedly higher in the TVT group as the sling is more obstructive when compared to the TOT, which has less obstructive hammock effect.

Novarra found that storage LUTS were less common after TOT tapes compared with the TVT.[15] Waltregny *et al*. found that 75% of patients with preoperative LUTS had a cure or improvement after TOT.[9]

Ballert *et al*. studied the effects of the TVT vs TOT on LUTS in women undergoing these procedures with SUI and mixed urinary incontinence. They found significant improvement in the storage symptoms in all patient groups. A significant reduction in LUTS was noted in 56.1% of patients.[19]

Unlike the TVT, following TOT, voiding dysfunction does not worsen, rather it improves postoperatively and de novo storage symptoms are infrequent.

## RETURN TO NORMAL ACTIVITY

The mean operation time is significantly less for the TOT when compared with the TVT.[7,8,13,18] This is also associated with a significantly faster return to activity TOT.[7]

## THIGH/GROIN PAIN AND VAGINAL INJURY/MESH EXTRUSION

Due to the passage of the TOT beneath the pubic ramus and an exit in the groin, vaginal injuries, thigh and groin pain occur more frequently with TOT when compared with TVT. In the Italian multicenter study, groin pain resolved in most cases within one month from surgery with analgesics alone.[8]

Abdel Fattah, in a randomized trial evaluating different routes of placement of the TOT, found no significant difference in postoperative pain with either route.[20] Mesh extrusion/erosion are known to occur in all procedures. Erosion rates using non-silicone-coated materials range from 0% to 1.6%.[14] All studies and reviews that investigated TOT alone or in comparison with the TVT showed significantly increased rates of vaginal injury/mesh extrusion with TOT.[7,10,14]

Erosion usually occurs in the first few weeks to months after insertion. Unrecognized vaginal lacerations occurring during TOT may predispose to subsequent mesh extrusion. Though the overall occurrence of vaginal wall injury (0.6%) during TOT was found to be very low,[16] the higher rate of vaginal lacerations with TOT when compared to TVT possibly accounts for the slightly higher rate of mesh extrusion with this route.[7,14]

Despite the fact that vaginal erosion/extrusion rates are higher with TOT tapes, this is relatively more benign, easily recognized and amenable to surgical treatment than erosions into the bladder and bowel, which is more dangerous and occurs with TVT insertion.

## RARE COMPLICATIONS

There are no reports about injury to major blood vessels or life-threatening bowel injury so far with TOT. Rare occurrence of necrotizing fasciitis and suspected obturator nerve injury have been reported with the TOT tape.[7,21,22]

## SUMMARY AND CONCLUSION

Considering the comparable success and satisfaction rates of TOT and reduced risk of intra-operative bladder injury, shorter operating time and quicker return to activities, we recommend TOT as the primary choice for the treatment of SUI. Severe life-threatening complications such as bowel and vascular injuries, which may occur with the TVT is completely avoidable with TOT.

Due to intrinsic differences in the trajectory of the sling, we suggest that the TOT be considered as the preferred option, particularly in women with previous scarring in the pelvis due to anterior pelvic reconstructive surgeries/colposuspension in which there is a greater than normal risk of bladder and bowel injury. Groin pain, vaginal erosion/extrusion of the tape though higher with TOT, is not life-threatening and is amenable to treatment and cure.

We therefore opine that TOT should be offered as the first surgical choice in women seeking relief from SUI.
